# CD8^+^ T Cells Are Required For Glatiramer Acetate Therapy in Autoimmune Demyelinating Disease

**DOI:** 10.1371/journal.pone.0066772

**Published:** 2013-06-21

**Authors:** Andrew F. Tyler, Jason P. Mendoza, Mihail Firan, Nitin J. Karandikar

**Affiliations:** Department of Pathology, University of Texas Southwestern Medical Center, Dallas, Texas, United States of America; University Hospital of Heidelberg, Germany

## Abstract

The exact mechanism of glatiramer acetate (GA, Copaxone®), an FDA-approved immunomodulatory therapy for multiple sclerosis (MS), remains unclear after decades of research. Previously, we have shown that GA therapy of MS induces CD8^+^ T cell responses that can potentially suppress pathogenic CD4^+^ T cell responses. Using a murine model of MS, experimental autoimmune encephalomyelitis (EAE), we now demonstrate that CD8^+^ T cells are necessary in mediating the therapeutic effects of GA. Further, adoptive transfer of GA-induced CD8^+^ T cells resulted in amelioration of EAE, establishing a role as a viable immunotherapy in demyelinating disease. Generation of these cells required indoleamine-2,3-dioxygenase (IDO), while suppressive function depended on non-classical MHC class I, IFN-γ, and perforin expression. GA-induced regulatory myeloid cells, previously shown to activate CD4^+^ regulatory T cells in an antigen-independent manner, required CD8^+^ T cells for disease suppression *in vivo*. These studies demonstrate an essential role for CD8^+^ T cells in GA therapy and identify their potential as an adoptive immunotherapeutic agent.

## Introduction

Multiple sclerosis (MS) is an immune-mediated disorder characterized by recurrent demyelinating lesions in the central nervous system (CNS) leading to an array of neurological symptoms [1]. The disease is characterized by accumulation of immune cells in the perivascular spaces of CNS parenchyma leading to inflammation and destruction of the myelin sheath surrounding neural axon processes [2]. Experimental autoimmune encephalomyelitis (EAE) is a well-established model of MS in which immunization with myelin antigens induces a characteristic T cell-mediated ascending paralytic disease [3]. CD4^+^ T cells are considered the major pathogenic population in EAE, as adoptive transfer of myelin-specific CD4^+^ cells to naïve mice leads to disease [4]. EAE is vital to the study of MS, and has led to the development of several current therapies [5].

Glatiramer acetate (Copaxone, GA) is an FDA-approved immunomodulatory therapy for MS. Originally developed as a substitute for myelin basic protein (MBP) in inducing paralytic disease in animals, the drug inhibited the induction of EAE and reversed signs of demyelination [6]. GA is composed of the four amino acids alanine, lysine, glutamic acid and tyrosine in a ratio resembling that of the hydrophobic, basic, acidic, and aromatic residues found in MBP. It has a molecular mass ranging from 6.7 to 11 kDa, with a mean of 7.7 kDa, and is approximately 60–70 amino acids in length. Despite extensive animal experimentation and decades of human use, the drug’s precise mechanism of action remains uncertain. GA incites robust Th2 CD4^+^ T cell responses that cross react with myelin-derived peptides and decrease pathogenic Th1 cell activation [7], [8]. It also induces CD4^+^CD25^+^ regulatory T cells through Foxp3 induction that suppress activation and proliferation of autoimmune CD4^+^ T cells [9]. While much of the focus on GA lies in its effects on CD4^+^ T cells, GA can also modulate several antigen-presenting cell populations, including B cells [10], microglia [11], monocytes/macrophages [12], [13], and dendritic cells [14], [15]. Although GA induces several complex phenotypes *in vivo*, its central action likely lies in its ability to be presented as an antigenic peptide within major histocompatibility complexes (MHC) [16].

We have observed that CD8^+^ T cells, commonly associated with anti-viral and anti-tumor effects, are highly involved during GA therapy. We previously demonstrated that while GA-specific CD8^+^ T cell response are deficient in MS patients compared to healthy controls, GA treatment greatly enhances CD8^+^ T cell responses to the drug [17]. Unlike polyclonal CD4^+^ T cell responses, these activated cells represent an oligoclonal expansion to a set of specific peptide epitopes, with dominant clones persisting over long periods of time [18]. Furthermore, we showed that these GA-induced CD8^+^ T cells could suppress CD4^+^ proliferation in response to anti-CD3 and peptide stimuli. This “GA-specific” suppression was mediated through non-classical major histocompatibility complex (MHC) class I molecules [19]. Thus, our results from studies in human MS suggest that a subset of CD8^+^ T cells adopt regulatory properties upon GA stimulus, leading to drug action by suppressing pathogenic cell populations.

To further probe the involvement of CD8^+^ T cells in the mechanism of GA in demyelinating disorders, we utilized the murine model to address their fundamental role in this process. We demonstrate here, for the first time, a critical and essential role for this subset of T cells in mediating the beneficial therapeutic effects of GA.

## Results

### Glatiramer Acetate Induces Proliferative Responses in CD8^+^ T Cells

**Figure 1:**
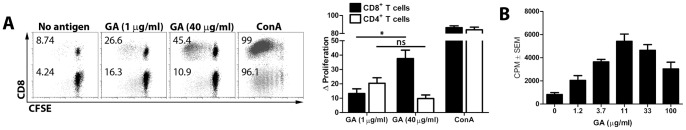
GA induces CD8+ T cell proliferative responses in mice. (A) Wild-type C57BL/6 mice were immunized with 2 mg GA/IFA. At day 20 post-immunization, bulk splenocytes and DLN cells were isolated, stained with CFSE, and cultured *in vitro* for 5 days with vehicle, GA (20 µg/ml), or concanavalin A (1 µg/ml). Data are gated for CD4+ and CD8+ T cells, with proportion of proliferating cells indicated. The bar graph represents cumulative data from multiple replicate experiments, represented as Δ proliferation (“No antigen” background proliferation subtracted). * = p<0.05, “ns” = not significant. (B) Wild-type C57BL/6 mice were immunized as in (A). Splenocytes and DLN cells were isolated and CD8^+^ T cells were purified by magnetic bead sorting. APCs were derived from spleens of OVA_323–339_/IFA-immunized mice and depleted of CD8^+^ T cells using anti-CD8 magnetic beads. GA CD8^+^ T cells were incubated in a 1∶4 ratio with APCs (1×10^6^ total cells/ml) for 4 days with increasing concentrations of GA. ^3^H-thymidine was added 24 hours before analysis. CPM are indicated on Y-axis. Data representative of over 5 replicates.

We first sought to determine if GA induces CD8^+^ T cell responses *in vivo* in mice similar to those observed in humans. C57BL/6 mice were immunized subcutaneously with GA in incomplete or complete Freund’s adjuvant (IFA or CFA), or administered daily subcutaneous GA following CFA immunization. Draining lymph nodes (DLN) were isolated 10 days post-immunization and a CFSE dilution-based proliferation assay was performed. GA induced antigen-specific recall responses in both CD8^+^ and CD4^+^ T cell populations (Fig. 1A shows IFA data as a representative). Notably, as GA concentration increased, CD8^+^ proliferation also increased while CD4^+^ proliferation began to decline. To confirm specificity of the CD8^+^ T cell response in the absence of GA-specific CD4^+^ T cells *in vitro*, magnetically purified CD8^+^ T cells derived from GA-immunized mice were co-incubated with irradiated T cell-depleted splenocytes derived from control OVA_323–339_/CFA-immunized mice as antigen-presenting cells. GA-specific CD8^+^ T cells were able to proliferate in the absence of CD4^+^ T cells and showed good dose-response curves (Fig. 1B).

### CD8^+^ T Cells are Necessary for the Action of Glatiramer Acetate

**Figure 2:**
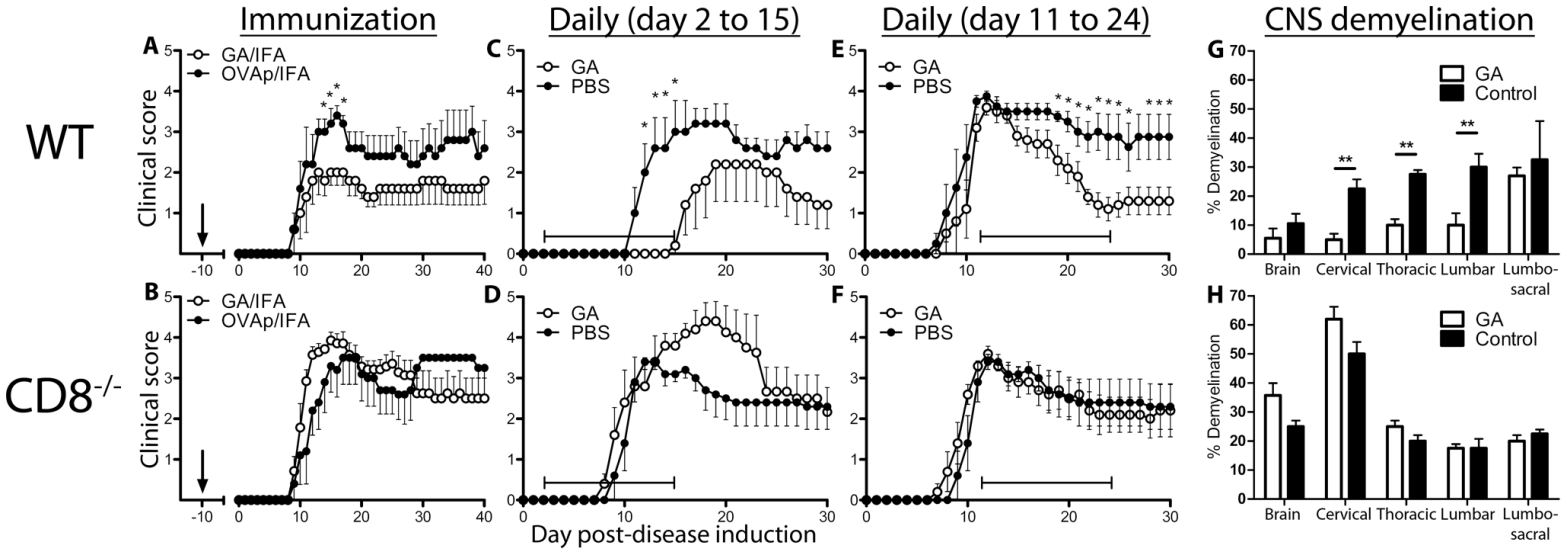
CD8+ T cells are required for GA action in ameliorating EAE. GA treatment was administered to wild-type (top row) or CD8^−/−^ (bottom row) mice by three treatment regimens: GA/IFA emulsion (2 mg GA) on day -10 (A,B), daily subcutaneous GA treatment (20 µg/mouse/day) from day 2 to 15 (C,D), or daily subcutaneous GA treatment from day 11 to 25 (E,F). EAE was induced on day 0. Mean disease scores ± SEM are indicated; representative of 5 replicate experiments. (G,H) Mice from (A) and (B) were euthanized 28 days after disease induction. Brains and spinal cords were processed, stained, and analyzed for percent demyelination. * = *p*<0.05, ** = *p*<0.01.

While CD8^+^ T cells are commonly associated with anti-viral and anti-tumor responses, several subsets have been linked with immune regulation in a host of autoimmune disorders, including models of diabetes [20], rheumatoid arthritis [21], systemic lupus erythematosus [22], and multiple sclerosis [23]–[26]. By utilizing mice deficient in CD8α, we could determine whether CD8^+^ T cells were necessary for GA-mediated inhibition of EAE. Thus, EAE was induced in wild-type C57BL/6 and CD8^−/−^ mice, which were subjected to three different treatment regimens: a subcutaneous injection of GA in IFA before disease induction (day -10) (Fig. 2, A and B), daily subcutaneous injections of GA after disease induction but prior to clinical signs of disease (day 2 to 15) (Fig. 2, C and D), and a therapeutic protocol during clinical disease (day 11 to 25) (Fig. 2, E and F). While each protocol was effective in wild-type mice, none of the protocols limited disease in CD8^−/−^ mice, and in some cases worsened symptoms. Examination of the CNS of these animals revealed lower levels of demyelination in the cervical, thoracic, and lumbar spinal cords of GA-treated wild-type mice compared to controls, whereas no such decrease was noted in CD8^−/−^ mice (Fig. 2, G and H).

### MHC Class I, IFN-γ, and Perforin are Required for GA Action in EAE

**Figure 3:**
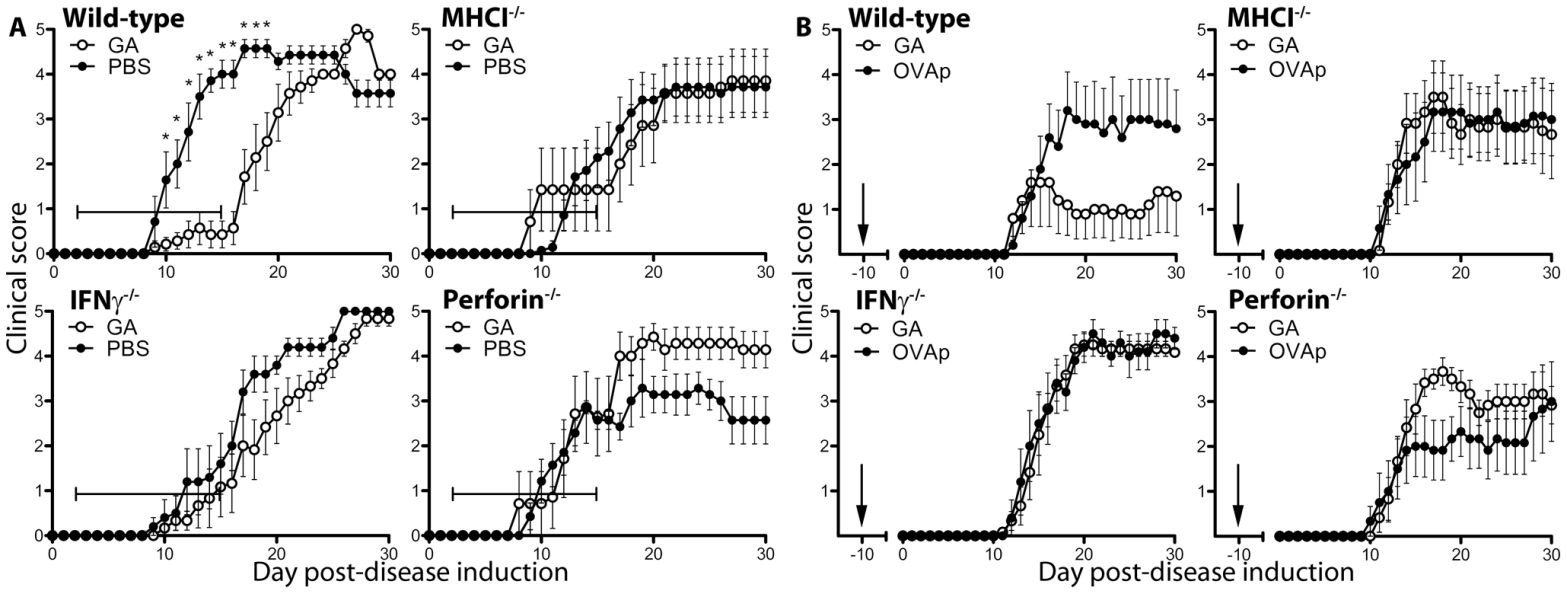
GA action in demyelinating disease is dependent on factors associated with CD8^+^ T cells. (A) EAE was induced in wild-type, β_2_microglobulin^−/−^ (MHCI^−/−^), IFN-γ^−/−^, and perforin^−/−^ mice. Mice were then treated with daily subcutaneous GA injection (20 µg/mouse/day) or vehicle (PBS) from day 2 to 15 after disease induction. (B) Wild-type, β_2_microglobulin^−/−^ (MHCI^−/−^), IFN-γ^−/−^, and perforin^−/−^ mice were immunized with GA (1 mg) in IFA ten days prior to disease induction and monitored for clinical disease. All data are representative of 3 replicate experiments.

To further explore our hypothesis that CD8^+^ T cells are required for GA action, we tested the drug on mice deficient in molecules associated with CD8^+^ T cell activity: MHC class I, interferon-γ (IFN-γ), and perforin. Cognate antigen presentation on MHC class I is required for TCR-mediated CD8^+^ T cell activation [27]. IFN-γ is produced during CD8^+^ T cell stimulation and can be an important immune regulatory factor [28,29]. In addition, GA treatment in MS patients induces CD8^+^ T cell-dependent IFN-γ production associated with drug action [30]. Like IFN-γ, perforin is typically linked to antiviral responses, but plays a vital role in the clearance of autoimmune T follicular helper (Tfh) cells in models of lupus [22]. To test the roles of these molecules in GA treatment, disease was induced in mice deficient in each of these molecules (MHCI^−/−^, IFN-γ^−/−^, perforin^−/−^). Mice then received either daily subcutaneous injections of GA from day 2 to 15 (Fig. 3A), or a single immunization of GA/IFA ten days prior to disease induction (Fig. 3B). While GA reduced signs of disease in wild-type mice, the drug was ineffective in reducing signs of disease in MHCI^−/−^, IFN-γ^−/−^, and perforin^−/−^ mice. Additionally, in some instances GA treatment worsened signs of disease, similar to observations in CD8-deficient hosts. Of note, GA is effective in ameliorating disease in IL4−/− as well as IL-10−/− mice, as demonstrated in prior reports [31].

### GA-specific CD8^+^ T Cells Ameliorate Signs of Demyelinating Disease and Depend on Non-classical MHC Class I for Activation and Disease Suppression

**Figure 4:**
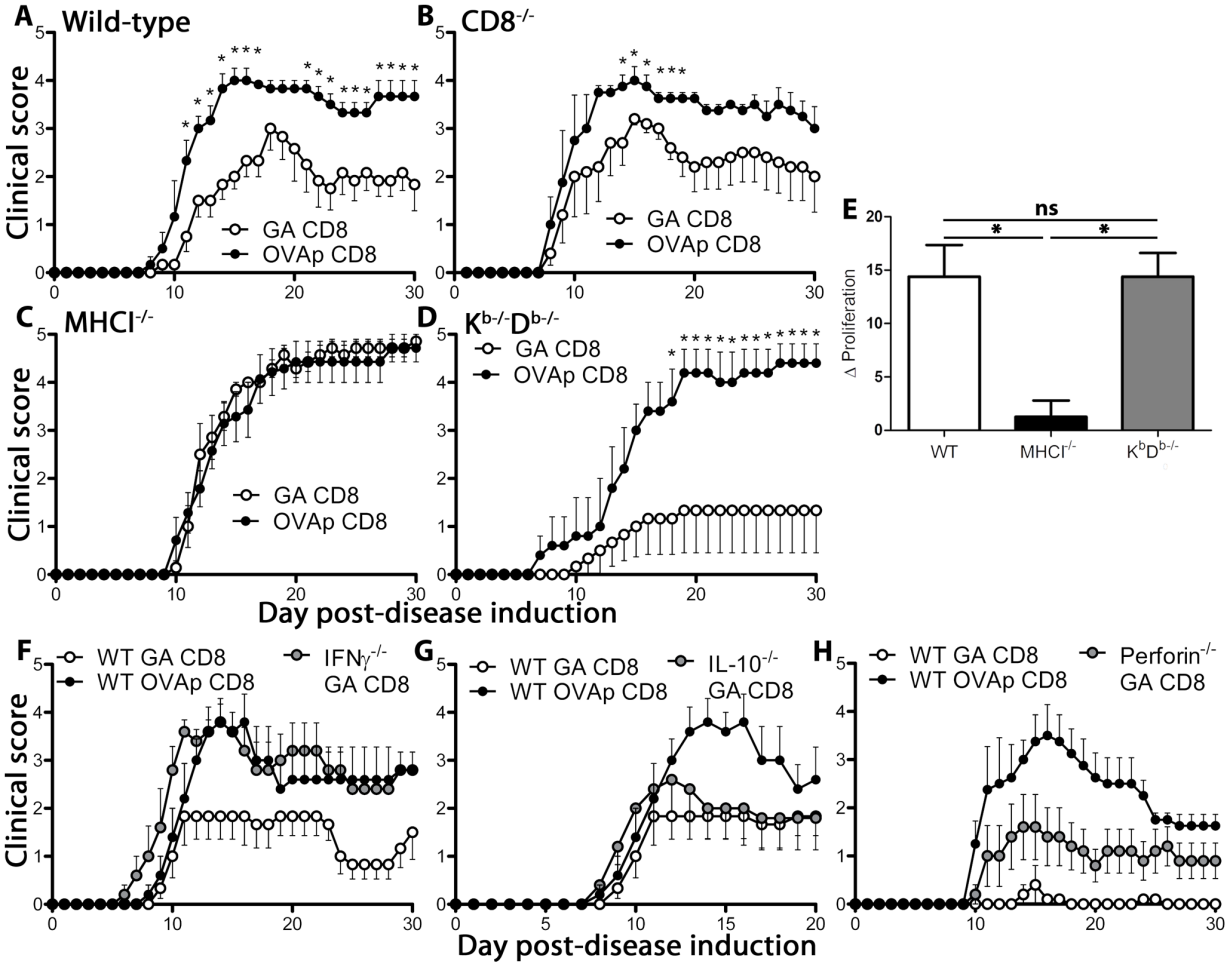
GA CD8^+^ T cells ameliorate demyelinating disease by a non-classical MHC class I-dependent mechanism requiring IFN-γ and perforin but not IL-10. (A–D) Donor wild-type C57BL/6 mice were immunized for 20 days with GA/IFA (2 mg) or OVAp/IFA (200 µg). Splenocytes and DLN cells were isolated and cultured *in vitro* for 3 days with antigen (20 µg/ml) and IL-2 (10 pg/ml). CD8^+^ T cells were purified by magnetic bead sorting and injected intravenously into wild-type (A), CD8^−/−^ (B), MHCI^−/−^ (β_2_microglobulin^−/−^) (C), and K^b−/−^D^b−/−^ (D) recipient mice one day prior to disease induction. (E) *Ex vivo* GA CD8^+^ T cells were isolated from GA/IFA-immunized mice and stained with CFSE, followed by incubation with APC derived from naïve wild-type, β_2_microglobulin^−/−^ (MHCI^−/−^) or K^b−/−^D^b−/−^ mice by depleting splenocytes of T cells using anti-CD3 magnetic beads. Replicate cultures were incubated with either vehicle or GA (20 µg/ml). Δ proliferation reflects difference in proportion of CFSE_low_ proliferating cells between GA and vehicle control cultures. * = p<0.05, “ns” = not statistically significant. (F-H) CD8^+^ T cells were obtained from GA/IFA or OVAp/IFA immunized wild-type (WT), IFN-γ^−/−^, perforin^−/−^, and IL-10^−/−^ mice as above and injected intravenously into wild-type recipient mice one day prior to EAE induction.

While CD8^+^ T cells are necessary for mediating GA action *in vivo*, it remained unclear whether GA-reactive CD8^+^ T cells are sufficient to suppress demyelinating disease. Whereas regulatory populations of auto-reactive CD8^+^ T cells are well-described [32–35], GA CD8^+^ T cells are notable in that they are activated by a synthetic molecule with an unclear natural analog. To test the regulatory capabilities of GA-induced CD8^+^ T cells, donor wild-type mice were immunized with GA or control antigen (OVAp) emulsified in IFA. After twenty days, DLN cells and splenocytes were isolated and restimulated *in vitro* with the corresponding antigen, followed by CD8^+^ T cell isolation. Cells were injected intravenously into wild-type or CD8^−/−^ recipient mice followed by disease induction one day later. In both wild-type and CD8^−/−^ mice, GA-reactive CD8^+^ T cells inhibited signs of clinical disease (Fig. 4, A and B).

Based on the earlier treatment data, we hypothesized that MHC class I was necessary to activate GA CD8^+^ T cells *in vivo* to induce disease suppression. To analyze the role of MHC class I in GA CD8^+^ T cell-mediated control of EAE, GA-reactive CD8^+^ T cells were adoptively transferred into MHCI^−/−^ and K^b−/−^D^b−/−^ recipient mice. While GA-reactive CD8^+^ T cells were not able to inhibit disease in MHCI^−/−^ mice, they were effective in K^b−/−^D^b−/−^ mice (Fig. 4, C and D), which still express non-classical MHC class I molecules such as Qa-1, the murine homolog of HLA-E [36]. Therefore, we sought to ascertain whether the suppressive ability of GA CD8^+^ T cells in K^b−/−^D^b−/−^ mice *in vivo* correlated with non-classical MHCI^−/−^ activation *in vitro*. To determine if GA-reactive CD8^+^ T cells are activated by MHC class I presentation *in vivo*, CD8^+^ T cells were isolated from GA-immunized mice and co-incubated with T cell-depleted splenocytes (T-APCs) from naïve mice that expressed MHC class I (WT), or lacked either general (MHCI^−/−^) or classical (K^b−/−^D^b−/−^) MHC class I expression. Compared to wild-type T-APCs, MHCI^−/−^ T-APCs could not support GA-specific CD8^+^ T cell proliferation, whereas proliferation was not affected when APCs lacked only classical MHC class I expression (Fig. 4E), suggesting restriction by non-classical MHC class I molecules.

### GA-specific CD8^+^ T Cells Require IFN-γ and Perforin, but not IL-10, to Inhibit Signs of Demyelinating Disease *in vivo*

**Figure 5:**
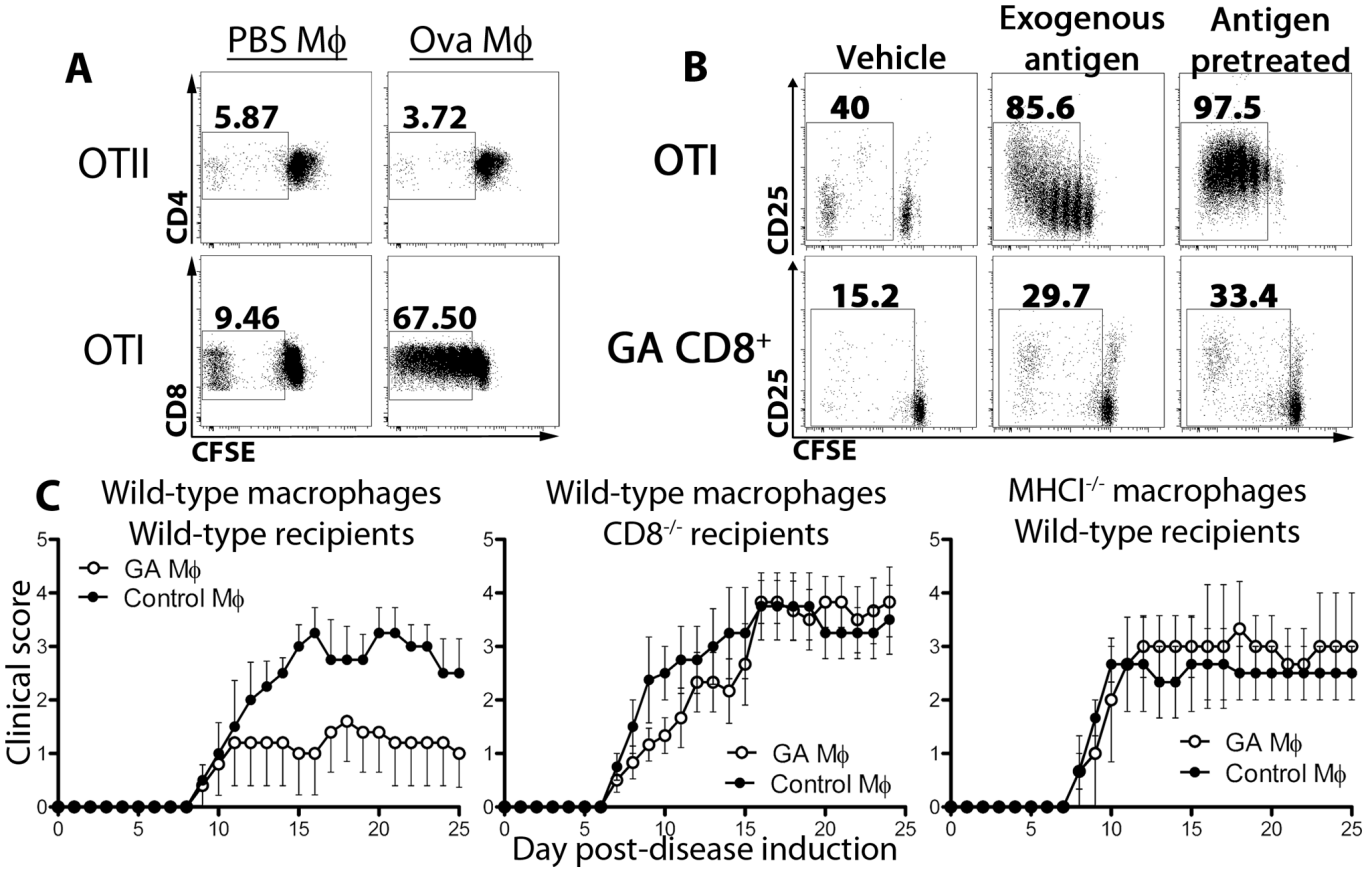
Suppressive bone-marrow macrophages induce antigen-specific proliferation in CD8^+^, but not CD4^+^, T cells and require CD8^+^ T cells and MHC class I for disease suppression. (A) Bone marrow cells were cultured *in vitro* for 6 days with M-CSF, IFN-γ, and hen egg ovalbumin (50 µg/ml) or vehicle (PBS). Cells were then combined *in vitro* with CFSE-stained OTI or OTII cells in a 1∶4 ratio (T cells : APCs) for 5 days. (B) Wild-type mice were immunized with GA/IFA (2 mg) for 20 days. Splenocytes were isolated and CD8^+^ T cells were purified by magnetic bead sorting and stained with CFSE. Bone-marrow monocytes were prepared as in (A) using ovalbumin or GA (50 µg/ml), followed by *in vitro* culture with OTI or GA CD8^+^ T cells, respectively, at a 1∶4 ratio (1×10^6^ total cells/ml) for 5 days with vehicle (PBS) or additional cognate antigen (ovalbumin or GA, 20 µg/ml). (C) Wild-type and MHCI^−/−^ bone-marrow cells were cultured with M-CSF (10 ng/ml), IFN-γ (100 U/ml), and GA (50 µg/ml) or vehicle (PBS). After 6 days GA- or control macrophages were injected into wild-type and CD8^−/−^ mice at peak disease. Wild-type recipient mice receiving MHC^−/−^ cells were depleted of NK cells by administration of anti-NK1.1 antibody (250 µg).

GA-induced CD8^+^ T cells express both IFN-γ and IL-10 (Fig. S1), signifying at least two different mechanisms of disease suppression. However, while earlier experiments revealed that IFN-γ and perforin are essential for GA-mediated disease suppression, IL-10, commonly associated with immunosuppression, is unnecessary for GA-mediated attenuation of disease [31]. The influence of IFN-γ, perforin, and IL-10 on GA CD8^+^ T cell-mediated EAE suppression was investigated by deriving GA-reactive CD8^+^ T cells from IFN-γ-, perforin-, and IL-10-deficient donor mice. These cells were transferred into wild-type recipient mice and compared to positive and negative controls. While IFN-γ-deficient cells lose regulatory ability, IL-10-deficient cells are able to suppress disease to similar levels as wild-type cells (Fig. 4, F and G). Perforin-deficient cells lose some of their regulatory capability, though it is not completely abrogated (Fig. 5H).

### Glatiramer Acetate-treated Monocytes Induce Regulatory CD8^+^ T Cell Populations

GA induces a “type 2”, anti-inflammatory phenotype in monocytes/macrophages both *in vivo* and *in vitro* thought to be responsible for disease amelioration [37]. However, mice depleted of phagocytic populations still respond to GA treatment (Fig. S2). Furthermore, as IL-10 is unnecessary for GA action, an anti-inflammatory phenotype may not offer a complete explanation for GA-mediated disease suppression. We therefore wanted to dissect the respective roles of monocytes/macrophages and CD8^+^ T cells in the drug action of GA and investigate the interactions between these cell types.

We first asked whether an anti-inflammatory phenotype could be induced in the absence of CD8+ T cells. Thus, wild-type and CD8^−/−^ mice were treated daily with subcutaneous injections of GA. After 6 days, splenic CD11b^+^CD11c^-^ cells were assayed for cytokine production. Though our earlier data illustrated the inability of GA to modulate disease in CD8^−/−^ mice (Fig. 2), a comparable shift toward an anti-inflammatory phenotype was induced by GA treatment in both wild-type and CD8^−/−^ cells (Fig. S3).

One manner in which GA-treated macrophages and CD8^+^ T cells could interact is the intracellular “carryover” of antigen into recipient mice. Although GA-treated monocytes do not activate CD4^+^ T cells in this manner [37], CD8^+^ T cell activation has not been investigated. We first tested this hypothesis by co-incubating ovalbumin- and vehicle-treated macrophages with CFSE-stained CD4^+^ OTII T cells and CD8^+^ OTI T cells. Ovalbumin was chosen as the activating agent due to a larger molecular weight and the presence of both CD4^+^ and CD8^+^ T cell epitopes, similar to GA. While OTII cells were not activated by macrophages that received ovalbumin during culture, OTI cells proliferated strongly (Fig. 5A). To test the idea of antigen carryover in a more disease-relevant setting, GA was also included in the next set of experiments. Thus, CFSE-stained OTI and GA-specific CD8^+^ T cells were incubated with vehicle-treated control macrophages plus media, control macrophages plus exogenous cognate antigen (ovalbumin or GA), or macrophages that were pretreated with antigen during differentiation. While macrophages were able to induce antigen-specific proliferation in both cells types in the presence of exogenous antigen, they activated antigen-specific CD8^+^ T cells more strongly after antigen-pretreatment, in the absence of additional exogenous antigen (Fig. 5B). It is essential to note that the macrophages were washed thoroughly prior to co-incubation to eliminate additional antigen in culture. Taken together, these results suggest that GA-incubated anti-inflammatory macrophages have the potential of carrying GA to stimulate CD8+ T cells *in vivo*.

To further evaluate the association between macrophages and CD8^+^ T cells in GA therapy, GA-treated bone marrow macrophages were adoptively transferred into wild-type and CD8^−/−^ mice at the peak of disease. While wild-type GA-treated macrophages decreased disease in wild-type recipients, disease was unaffected in CD8^−/−^ mice (Fig. 5C). Therefore, it is possible that GA-treated macrophages activate CD8^+^ T cells after adoptive transfer *in vivo* via MHC class I/TCR interactions. To test this, GA-treated MHCI-deficient macrophages were adoptively transferred into wild-type recipient mice. NK cell depletion was used to avoid killing of the macrophages. MHC I-deficient cells did not suppress signs of disease, in contrast to wild-type cells, suggesting that GA-reactive CD8^+^ T cells are downstream effectors of GA macrophage-mediated disease suppression (Fig. 5C).

### IDO is Required for the Action of GA-induced Regulatory CD8^+^ T Cells

**Figure 6:**
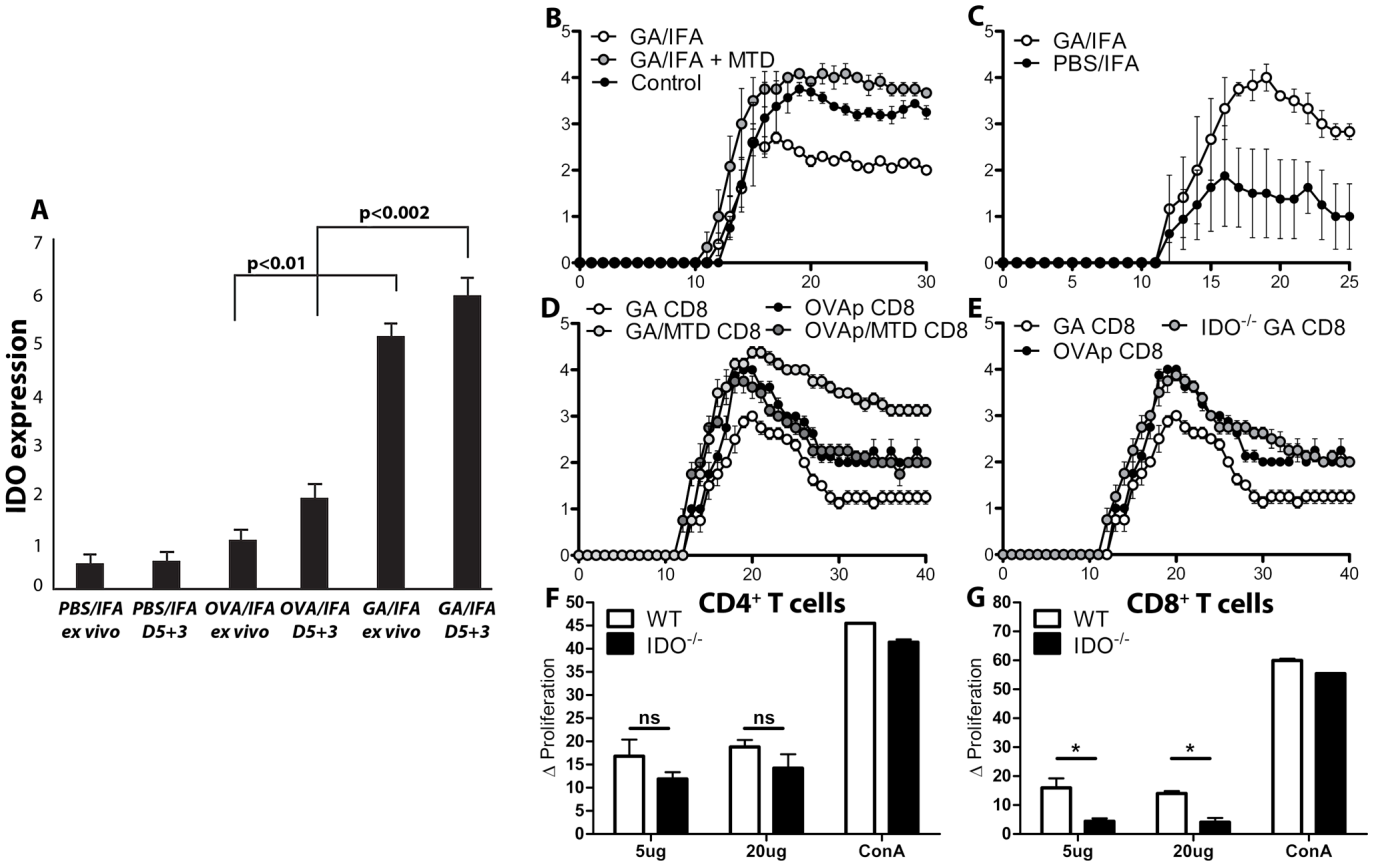
GA induces indoleamine-2,3-dioxygenase expression leading to suppressive behavior in CD8^+^ T cells. (A) Wild-type C57BL/6 mice were subcutaneously immunized with PBS/IFA, OVA/IFA (200 µg), or GA/IFA (2 mg). At day 5, DLNs were isolated and T cells were depleted by anti-CD3 magnetic beads. RNA was isolated from the remaining cells and IDO transcript levels were quantified by rtPCR. (B) Wild-type C57BL/6 mice were immunized with GA/IFA, GA+MTD/IFA, or vehicle one day prior to disease induction with MOG_35–55_. (C) MOG_35–55_ EAE was induced in wild-type or IDO^−/−^ C57BL/6 mice. (D) Donor wild-type C57BL/6 mice were immunized with GA/IFA, GA+MTD/IFA, OVAp/IFA, or OVAp+MTD/IFA. At day 20, CD8^+^ T cells were isolated after 3 day *in vitro* culture and adoptively transferred into wild-type C57BL/6 recipient mice. Disease was induced with MOG_35–55_. (E) As in (D), but including IDO^-./−^ donor mice. (F, G) Wild-type and IDO^−/−^ mice were immunized with GA/IFA (2 mg) for 10 days. DLN cells were isolated and stained with CFSE, followed by 5 day *in vitro* culture. “ns” = not statistically significant, * = p<0.05.

Foreign antigens typically provoke strong immune responses, particularly when administered with adjuvant. Nonetheless, some antigens prove tolerogenic by inducing cellular factors that lead to anti-inflammatory cytokine secretion and the induction of regulatory T cells [38]. One of these factors is indoleamine-2,3-dioxygenase (IDO). IDO is a tryptophan-metabolizing enzyme that is strongly up-regulated in lymphoid tissues by pro-inflammatory molecules such as LPS and IFN-γ [39]. IDO^+^ monocyte-derived macrophages are able to inhibit T cell proliferation in vitro, and IDO expression in IFN-γ-activated DCs inhibits priming to T cell antigens [40,41]. Therefore, we wanted to discern the role of IDO in GA action. Mice were immunized with GA/IFA. Five day post-immunization, DLNs were isolated and depleted of T cell with anti-CD3 magnetic beads. IDO transcripts were measured either directly ex vivo or after three day restimulation culture. While vehicle- and control antigen-immunized mice displayed no significant changes in IDO expression, GA-treated cells greatly increased levels of IDO transcript up to sixfold higher (Fig. 6A).

IDO induction can lead to inhibition of pathogenic immune responses; therefore, we hypothesized that IDO may be responsible for some of the immunosuppressive effects of GA treatment. To determine IDO’s effect on GA-mediated suppression of EAE, mice were immunized with GA in adjuvant with or without the addition of 1-methyltryptophan-D (MTD), an inhibitor of IDO enzymatic activity *in vivo*, followed by EAE induction. Whereas mice that received GA displayed reduced levels of disease, mice treated with both GA and MTD exhibited no decrease in symptoms (Fig. 6B). Similarly, GA had no protective effect in IDO^−/−^ mice, and instead increased signs of disease (Fig. 6C), similar to that observed in CD8- or perforin-deficient mice.

We next wanted to establish IDO’s role in GA-specific CD8^+^ T cell-mediated suppression. Donor mice were immunized with GA or control antigen with or without MTD. CD8^+^ T cells were purified and injected into wild-type recipient mice one day prior to disease induction. GA CD8^+^ T cells from donor mice that had received MTD no longer inhibited disease progression (Fig. 6D), and instead increased disease severity above that of control-treated mice. Similarly, GA CD8^+^ T cells from IDO^−/−^ were also unable to suppress signs of EAE (Fig. 6E).

IDO insufficiency resulting in the inability of GA-specific CD8^+^ T cells to regulate demyelinating disease may be due to a decrease in the activation of these cells. To test this aspect of IDO in relation to GA CD8^+^ T cells, wild-type and IDO^−/−^ mice were immunized with GA/IFA. After twenty days splenocytes were isolated and stained with CFSE to determine the antigen-specific activation of T cell populations. While no differences were observed in the proliferation ability of CD4^+^ T cells, the activation of CD8^+^ T cells from IDO^−/−^ mice was greatly reduced (Fig. 6, F and G).

### GA CD8^+^ T Cells Access the CNS during Autoimmune Neuroinflammation, Reduce Ex vivo Neuroantigen-specific Proliferation of CD4^+^ T Cells, and are Necessary to Induce CD4^+^CD25^+^Foxp3^+^ Regulatory T cells *in vivo*

**Figure 7:**
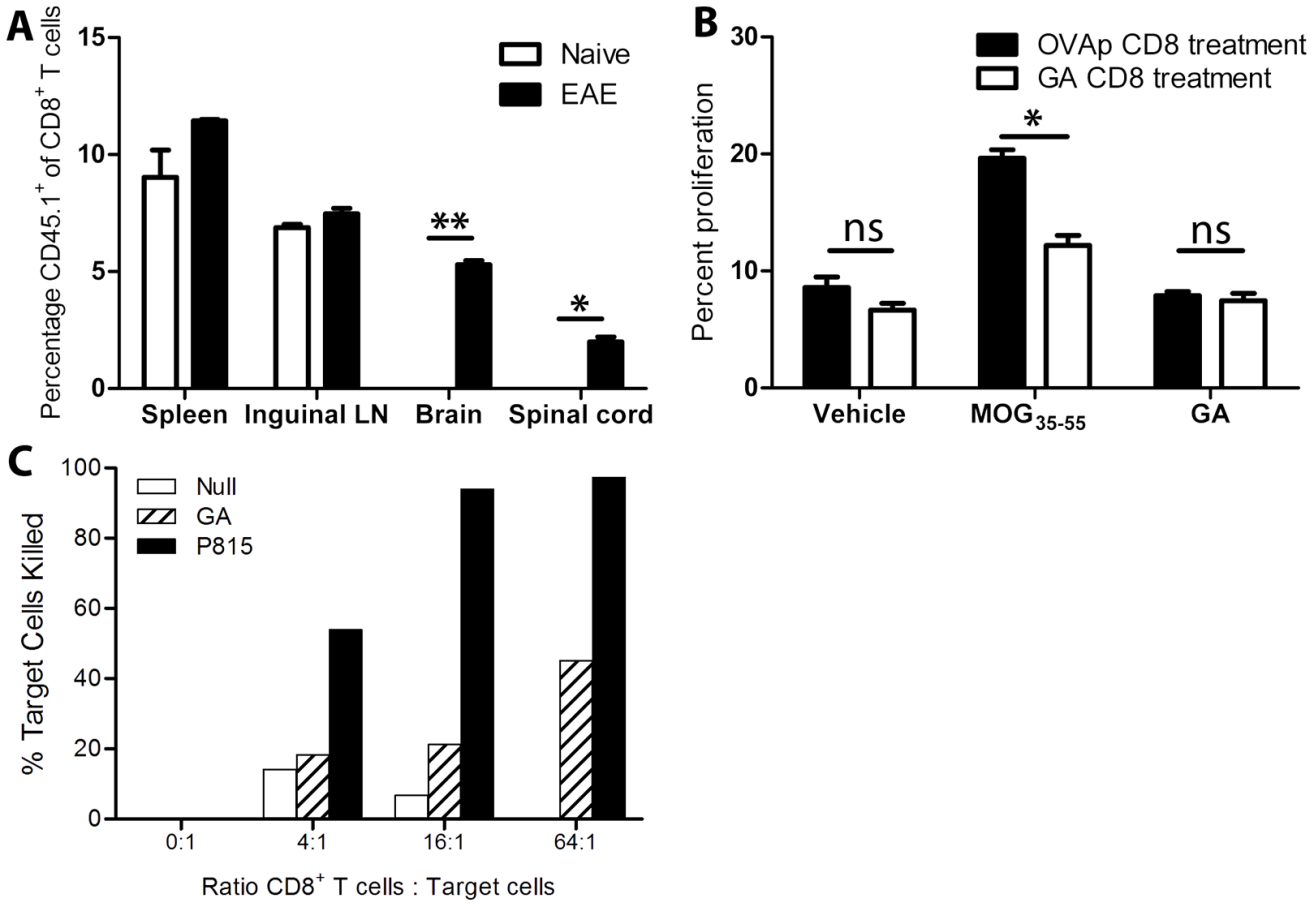
GA CD8^+^ T cells access the central nervous system during autoimmune inflammation, inhibit *ex vivo* neuroantigen-specific CD4^+^ T cell proliferation, and are cytotoxic toward GA-loaded target cells. (A) CD45.1^+^ GA CD8^+^ T cells were injected into naïve or EAE-induced recipient mice (at peak disease). Twenty-four hours post-transfer, tissues were processed and stained for CD45.1^+^CD8^+^ T cells. * = p<0.05, ** = p<0.01. (B) Splenocytes were isolated at day 25 post-disease induction from mice that received control- vs. GA CD8+ T cells. They were stained with CFSE, and assayed for proliferative CD4 T cell responses to vehicle, MOG_35–55_ (20 µg/ml), or GA (20 µg/ml). “ns” represents not statistically significant. (C) Twenty days post-GA/IFA immunization, CD8^+^ T cells from draining lymph nodes and spleens were purified by magnetic bead selection and subjected to an *in vitro* cell-mediated cytotoxicity assay, using either GA-loaded target cells or anti-CD3+P815 (positive control). % killing is shown on Y-axis with increasing E:T ratios on X-axis.

While regulatory T cells often exert immunomodulatory effects by trafficking to secondary lymphoid organs and interacting with antigen-presenting cells as well as other T cells, in some situations these cells localize in a tissue-specific manner [42,43]. To determine if GA CD8^+^ T cells could enter the CNS in order to suppress disease, GA CD8^+^ T cells were derived from congenic CD45.1^+^ mice and transferred into naïve and EAE-induced recipients at peak disease. Twenty-four hours post-transfer, peripheral and CNS tissues were isolated and assayed for the presence of donor cells. While cells were found peripherally in both hosts, GA CD8^+^ T cells only migrated to the CNS during active inflammation (Fig. 7A).

GA-reactive CD8^+^ T cell-mediated may suppress disease by reducing the expansion of pathogenic, CNS-specific CD4^+^ T cells, thereby inhibiting the inflammatory response and leading to decreased disease levels. In order to determine the effects of GA CD8^+^ T cells on pathogenic, neuroantigen specific CD4^+^ T cells, splenocytes from wild-type mice that had received GA or control CD8^+^ T cells were isolated twenty-five days after disease induction and analyzed for proliferative responses toward the immunizing CNS auto-antigen (MOG_35–55_). While background and non-cognate antigen proliferation levels were similar, cells from protected mice showed far less proliferation in response to CNS auto-antigen than did those from unprotected mice (Fig. 7B). This inhibition could be due to the death of CNS-reactive CD4^+^ T cells induced by cytolytic GA CD8^+^ T cells responding to peptide epitopes resembling GA. To test this possibility, target splenocytes were stained with CFSE and loaded with vehicle or GA and cultured with *in vitro*-activated GA CD8^+^ T cells. After 4 hours, GA-loaded target cells demonstrated dose-dependent cell death in response to GA-specific CD8^+^ T cells (Fig. 7C).

**Figure 8:**
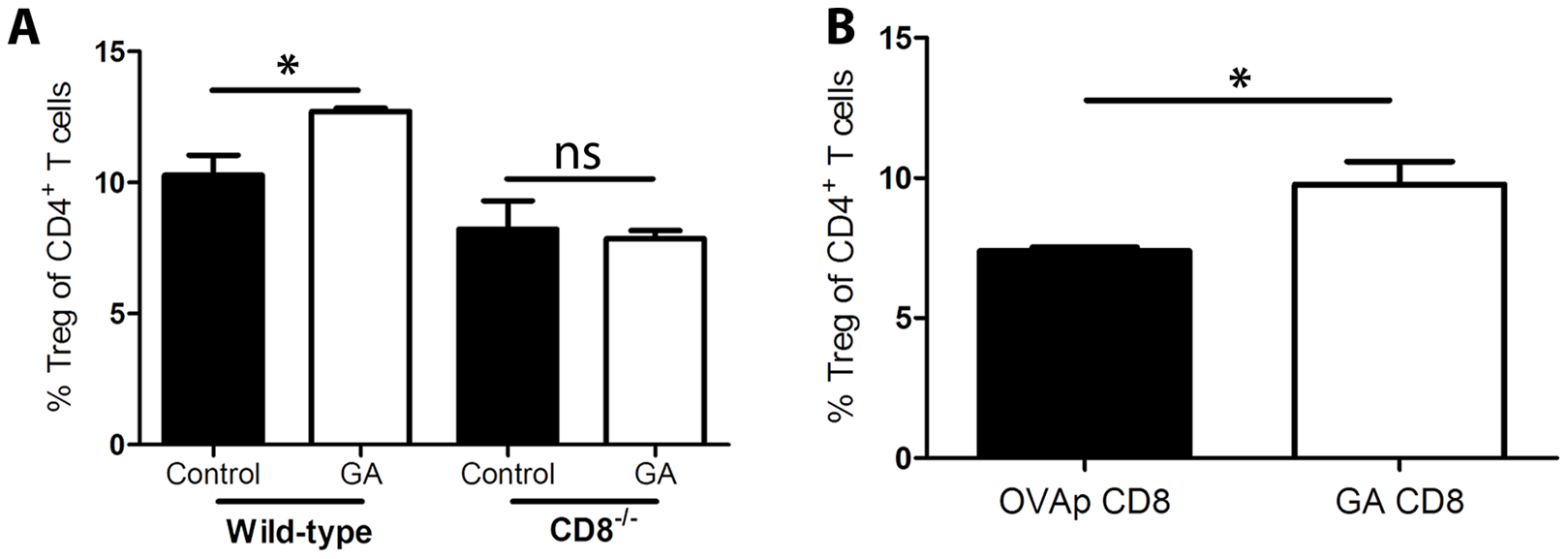
GA CD8^+^ T cells are necessary to induce CD4^+^ Tregs *in vivo* **.** (A) GA- or PBS-treated (control) bone marrow macrophages were adoptively transferred into wild-type or CD8^−/−^ recipient mice at peak disease. Twenty five days post-EAE induction, splenic CD4^+^CD25^+^Foxp3^+^ regulatory T cells were quantified by flow cytometry. (B) CD8^+^ T cells derived from GA/IFA- or OVAp/IFA-immunized mice were transferred into wild-type hosts one day prior to EAE induction. After 25 days splenic CD4^+^CD25^+^Foxp3^+^ regulatory T cells were stained and quantified by flow cytometry.

CD4^+^ Tregs are thought to play a major role in the macrophage-mediated inhibition of demyelinating disease [37]. Therefore, if CD8^+^ T cells are required for the action of GA-treated macrophages in decreasing signs of EAE, they may also be necessary for the induction of Tregs *in vivo*. To test this hypothesis, spleens were isolated from wild-type and CD8^−/−^ mice that had received GA-treated monocytes and evaluated for CD4^+^CD25^+^Foxp3^+^ Tregs. While Tregs significantly increased in wild-type mice receiving GA monocytes compared to controls, no rise was observed in CD8^−/−^ mice (Fig. 8A). We further tested whether GA CD8^+^ T cells were sufficient to induce CD4^+^ Tregs. Splenocytes isolated from mice that received GA-reactive CD8^+^ T cells were compared to those of mice treated with control cells to evaluate changes in Treg populations. The cells derived from GA CD8^+^ T cells-treated mice showed a significant increase in the percent of Tregs (Fig. 8B).

## Discussion

Multiple sclerosis is a devastating disease, responsible for a drastic decrease in the quality of life for millions of patients worldwide. Current treatments for the disease are focused on tempering a run-away immune system - unfortunately, many of these drugs have dangerous side effects that limit their widespread use. Glatiramer acetate is an indispensable FDA-approved therapy for the treatment of multiple sclerosis, due to its efficacy and lack of major side effects. Due to its success in MS, the drug is currently being tested in a variety of other autoimmune disorders as well as graft rejection [44–46]. However, the incomplete understanding of its mechanism of action exhibits a failure to fully appreciate the drug’s potential. Our findings investigate a heretofore under-appreciated player in the drug’s mechanism, the CD8^+^ T cell. Earlier work by our group revealed that GA induces proliferative responses in CD8^+^ T cells and treatment with the drug increases these responses in MS patients [17]. These cells in turn can then suppress the proliferation of CD4^+^ T cells in an antigen-specific manner, demonstrating a possible role for these cells in immune regulation [19]. The current study develops these initial findings into a more comprehensive mechanism, underscoring the relevance of these cells in the mechanism.

To our knowledge, the present work is the first to demonstrate that CD8^+^ T cells are absolutely required for GA action *in vivo*. CD8^+^ T cells are vital for both GA-mediated decreases in clinical disease and demyelination, establishing CD8^+^ T cells’ critical role in the drug’s action. In addition, effector molecules associated with CTL function such as MHC class I, IFN-γ, and perforin are crucial for drug activity. This indicates that GA directly activates pathways that induce CD8^+^ T cell responses and their ensuing regulatory capabilities. However, it remained unclear after the induction of regulatory CD8^+^ T cells whether the drug’s further presence was required for disease suppression. By adoptive transferring GA-induced CD8^+^ T cells into diseased mice and reducing signs of clinical disease, we demonstrate that pre-activated GA CD8^+^ T cells maintain their regulatory phenotype outside of the drug’s presence, requiring activation by MHC class Ib *in vivo*. In addition, while IFN-γ is absolutely required for GA CD8^+^ T cell-mediated disease suppression, perforin expression appears to be only partially responsible, and may therefore be more important for other regulatory mechanisms induced by GA, such as NK cell-mediated cytolysis of pathogenic cell populations [47].

Mechanistic studies of GA have elucidated antigen-presenting cells as possible targets of drug action. Our studies expand upon these findings by interrogating the interaction between GA-treated macrophages and CD8^+^ T cells. Our data show that the type 2 phenotype is induced in monocytes derived from both wild-type and CD8-deficient mice. However, while the drug is effective in reducing disease in wild-type mice, it is not effective in CD8-deficient mice. This leads us to believe that the type 2 phenotype is insufficient in explaining GA’s ability to reduce signs of demyelinating disease. Furthermore, GA-treated macrophages do not reduce signs of EAE in mice lacking CD8^+^ T cells, nor can these cells suppress disease if they are lacking MHCI. This suggests that CD8^+^ T cells act as an effector population induced by GA-treated macrophages. One way by which the macrophages could induce CD8^+^ T cells is “carry-over” of antigen from *in vitro* culture into recipient animals. Although CD4^+^ T cells cannot be activated in that manner, our data clearly demonstrate CD8^+^ T cell proliferation from both OVA- and GA-treated macrophages in an antigen-specific manner without additional antigen. This leads to the hypothesis that GA-treated macrophages induce regulatory populations of GA-specific CD8^+^ T cells *in vivo*. The downstream action of CD8^+^ T cells is confirmed by the observation that while macrophages induce CD4^+^CD25^+^Foxp3^+^ regulatory T cells in wild-type hosts, that ability is lost in CD8-deficient mice. GA-treated macrophages are therefore dependent on CD8^+^ T cells as effectors in reducing signs of neuroinflammation, indicating a mulit-step pathway toward disease amelioration.

While IFN-γ is primarily considered an inflammatory mediator, it can also induce anti-inflammatory and immunoregulatory effects both *in vivo* and *in vitro*. One of these effects is the induction of IDO, a tolerogenic protein expressed in a number of antigen-presenting cell populations. Our data show that IDO is clearly required for GA action *in vivo*. In addition, IDO is necessary for the induction of regulatory behavior in GA CD8^+^ T cells. Moreover, GA CD8^+^ T cells require the expression of IDO for efficient activation and proliferation, whereas CD4^+^ T cells do not. Finally, GA itself can induce expression of IDO *in vivo*, suggesting positive reinforcement between IDO expression and GA-specific proliferation.

Our data lead to a novel mechanism explaining GA action in regulating autoimmune disease. We propose a model where GA acts on antigen-presenting cells and is directly presented to CD8^+^ T cells to induce GA-specific regulatory CD8^+^ T cell activation, proliferation, and IFN-γ and perforin expression. This leads to the induction of IDO, which leads to further activation and proliferation and stabilization of the regulatory phenotype in these cells. GA CD8^+^ T cells go on to recognize target cells expressing a molecular mimic of GA, reducing pathogenic APCs or CD4^+^ T cells directly either through cell-mediated cytotoxicity or the induction of regulatory factors. Both of these mechanisms can lead to a decrease in auto-antigen specific proliferation and a rise in CD4^+^CD25^+^Foxp3^+^ regulatory T cells, which themselves can then synergistically contribute to the immunoregulatory phenotype.

GA is an important therapy for MS despite emergence of several others in recent years, and its clinical use continues to expand as new uses are found for the drug. The current work represents a novel mechanism for a synthetic drug enjoying widespread use in demyelinating autoimmune disease. Through future studies, the proposed paradigm will help determine the functional moiety of GA, a random polypeptide for which the exact molecular interactions governing the regulatory phenotype are unknown, by providing cellular targets for isolation and analysis. Furthermore, it demonstrates the viability of an autologous cell transfer approach for adoptive immunotherapy, unveiling new strategies in the management of MS and other disorders.

## Materials and Methods

### Mice

Female C57BL/6 mice aged 6–8 weeks were purchased from Taconic Farms and the UT Southwestern Mouse Breeding Core Facility. CD8α^−/−^, perforin^−/−^, IFN-γ^−/−^, β_2_microglobulin^−/−^, and B6.SJL mice were purchased from Jackson Laboratories. IDO^−/−^ mice were a kind gift from Dr. Andrew Mellor of the Medical College of Georgia. All mice were housed and bred in the UT Southwestern Medical Center Animal Resource Center under pathogen-free conditions and maintained in a 12/12-hour light/dark cycle in temperature-controlled facilities. Animals were humanely euthanized by CO_2_ asphyxiation. All studies were in accordance with the regulations of the Institutional Animal Care and Use Committee of UT Southwestern Medical Center (UTSW IACUC) (Protocol Number: 2010–0037).

### CFSE-based Proliferation Assay

Carboxyfluorescein succinimidyl ester (CFSE) dilution assays were performed as previously described [18]. Briefly, cells were suspended at 1×10^6^ cells/ml in PBS and incubated at 37°C for 7 minutes with 0.25 µM carboxyfluorescein diacetate N-succinimidyl ester (CFDA-SE). Cells were then washed 3X with 10% fetal bovine serum (FBS, Gemini Bio-Products) in PBS. Cells were then resuspended in complete media (RPMI 1640 supplemented with 10% FBS, L-glutamine (2 mM), penicillin/streptomycin (100 IU/ml/100 µg/ml), HEPES (10 µM), sodium pyruvate (1 mM), non-essential amino acids (all from Mediatech), and β-mercaptoethanol (50 µM) (Sigma)) and incubated for 120 hours at 37°C and 5% CO_2_. Proliferation was considered significant if the Δ Proliferation (percent proliferation of sample – percent background proliferation) was >1% and the stimulation index (percent proliferation of sample/percent background proliferation) was >2.

### Flow Cytometry

Cells were washed with FACS buffer (PBS with 1% bovine serum albumin (BSA) and 0.1% sodium azide). For proliferation assays, cells were stained with anti-TCRβ (H57-597), anti-CD8α (53–6.7), anti-CD4 (RM4-5), and anti-CD25 (PC61) fluorescent antibodies (BD Biosciences). Tregs were stained using a Foxp3/transcription factor staining buffer set (eBioscience) using anti-CD4, anti-CD25 (3C7, BD Biosciences), and anti-Foxp3 (FJK-16s, eBioscience) antibodies. Flow cytometric data were acquired on a BD LSR II running FACSDiva software. Data were analyzed by FlowJo (TreeStar).

### Thymidine-incorporation Assay

Lymph node cells and/or splenocytes were harvested from GA-, OVA-, or MOG-immunized mice. Magnetically sorted CD8^+^ T cells were cultured in 96-well plates at a concentration of 2.5×10^5^ cells per well for CD8+ cells in 200 µl media. Irradiated splenocytes (3000 rad) from OVAp/IFA-immunized mice were used as antigen presenting cells (APC) at a ratio of 1∶4 CD8^+^ T cells to APCs. After 72 hours in culture, cells were pulsed with 0.5 µCi/well of [^3^H]methyl-thymidine for 18 hours. Cells were then harvested and analyzed for incorporation of radioactivity using a Wallac Betaplate counter (Wallac).

### EAE Induction

EAE was induced in C57BL/6 mice by subcutaneous immunization with 200 µg of MOG_35–55_ (MEVGWYRSPFSRVVHLYRNGK, Protein Chemistry Technology Center, UT Southwestern) in emulsified CFA (Difco Laboratories) supplemented with 4 mg/ml M. tuberculosis strain H37Ra (Difco) followed by intraperitoneal injection of 250 ng of Bordetella pertussis toxin (Difco) in phosphate-buffered saline (PBS) at the time of and 2 days after immunization. In the SJL model EAE was induced by subcutaneous injection of 100 µg of PLP_139–151_ (HSLGKWLGHPDKF, Protein Chemistry Technology Center, UT Southwestern) in emulsified CFA supplemented with 4 mg/ml M. tuberculosis H37Ra. Clinical disease severity was monitored daily and scored according to the following scale: 0– no clinical disease, 1– limp tail, 2– hind limb weakness, 3– severe hind limb weakness and/or partial hind limb paralysis, 4– complete hind limb paralysis, 5– front limb weakness/moribund/death. Upon reaching a clinical score of 2 special care was given to animals, including delivery of moist food in a dish on the cage floor as well as removal of more severely diseased animals into separate cages. All animal experiments were repeated at least three times.

### Glatiramer Acetate

Glatiramer acetate (Copaxone, GA, Teva Neuroscience) was graciously donated by the UT Southwestern Multiple Sclerosis Center. Mice were immunized by subcutaneous injection of either GA (2 mg) or hen ovalbumin residues 323–339 (OVAp, ISQAVHAAHAEINEAGR, Protein Chemistry Technology Center, UT Southwestern) emulsified in 200 µl incomplete Freund’s adjuvant (IFA, Difco). For daily regimens, GA was subcutaneously administered at 20 µg per mouse per day in 100 µl PBS. For experiments involving IDO inhibition 1-methyltryptophan-D (MTD, Sigma) (5 mg/ml) was included in the emulsion.

### Histology

Mice were humanely euthanized, perfused with saline, and brain and spinal cord sections were given to the UT Southwestern Neuropathology histology core for processing and paraffin-embedment. Sections were stained with luxol fast blue (LFB) to visualize demyelination. Demyelination was quantified by trained neuropathologists in a blinded manner.

### Adoptive Transfer of Regulatory CD8^+^ T Cells

Donor mice were subcutaneously immunized with GA or OVAp emulsified in IFA, as above. Twenty days post immunization, splenocytes were harvested and stimulated *in vitro* with GA or OVAp (20 µg/ml) and hIL-2 (10 pg/ml) in complete media at 7.5×10^6^ cells/ml for 72 hours at 37°C in 5% CO_2_. Live cells were separated by density gradient (Lympholyte-M, Cedarlane Labs). CD8^+^ T cells were purified with CD8α (Ly-2) microbeads (Miltenyi Biotec). Purity was >90% by flow cytometry. Cells (5×10^6^) were transferred intravenously.

### *In vitro* Induction of Suppressive Macrophages

Suppressive macrophages were induced as previously described [37]. Briefly, bone marrow was isolated from wild-type or MHCI^−/−^ C57BL/6 mice and incubated at 1×10^6^ cells/ml in six-well plates (5 ml per well) in complete macrophage media (DMEM/F12 (Mediatech) supplemented as above plus 10 ng/ml M-CSF (Sigma)) including 100 U/ml IFN-γ with or without 50 µg/ml GA. Half of culture supernatants were replace with media and IFN-γ on day 3. On day 6 cells were washed twice with ice cold PBS and scraped off of plates using a rubber policeman. Cells were either transferred to recipient mice (1×10^6^ per mouse) or used in *in vitro* studies. Mice receiving MHCI^−/−^ cells were treatment intraperitoneally with anti-NK1.1 antibody (250 µg/injection) 7 and 1 days before, and every 7 days after, disease induction.

### *In vitro* Proliferation of CD8^+^ T Cells by Monocytes

CD4^+^ T cells from OTII mice and CD8^+^ T cells from naïve OTI and GA-immunized mice were isolated by magnetic bead separation. OVA macrophages were derived as above, replacing GA with hen egg ovalbumin (50 µg/ml, Sigma). Cells were stained with CFSE and incubated in a 1∶4 ratio (CD8^+^ T cells to monocytes) for five days. Exogenous antigen was added at 20 µg/ml. Cells were stained and analyzed as above.

### Quantitative Real-time PCR

Total RNA was extracted from cells using RNeasy Mini Kit (Qiagen) followed by reverse transcription using Superscript II reverse transcription kit (Qiagen). Quantitative real-time PCR assays were performed using Brilliant SYBR Green QPCR Master Mix on a MX3000p thermocycler. The following primer pairs were used: β-actin: (F) GTGGGCCGCTCTAGGCACCAA, (R) CTCTTTGATGTCACGCACGATTTC; IDO: (F) CACTGATACGCCTGAGTG, (R) GTGAGCGCTGAATCGAAA. IFN-γ: (F) AGCAACAGCAAGGCGAAAAA, (R) AGCTCATTGAATGCTTGGCG; TNF-α: (F) CATCTTCTCAAAATTCGAGTGACAA, (R) TGGGAGTAGACAAGGTACAACCC; IL-10: (F) CAGAGCCACATGCTCCTA, (R) GGAGTCGGTTAGCAGTATG; IL-6: (F) CTGATGCTGGTGACAACCAC, (R) ACCAGAGGAAATTTTCAATAGGC; TGF-β: (F) CACTGATACGCCTGAGTG; (R) GTGAGCGCTGAATCGAAA.

### Preparation of Clodronate- and PBS-loaded Liposomes

Liposomes were prepared as previously described [48]. Briefly, cholesterol (8 mg) (Sigma) was dissolved in chloroform (10 ml), followed by addition of 0.86 ml of a solution of phosphatidylcholine (Sigma) in chloroform (100 mg/ml). Low vacuum rotovap was applied to remove chloroform. The remaining phospholipid film is dispersed in 10 ml of either PBS (for control liposomes) or a 0.6 M solution of dichloromethylene-bisphosphonate (clodronate, Sigma) in water, and kept under nitrogen for 2 hours at room temperature. Solutions were sonicated in a waterbath for 3 minutes and swelled under nitrogen overnight at 4°C. Prior to use, liposomes are centrifuged at 10000×g, collected, then further washed and resuspended in PBS.

### Depletion of Phagocytic Macrophages

Mice were intraperitoneally treated with PBS- or clodronate-loaded liposomes (200 µl) beginning on day -3 and continuing every four days. Disease was induced by subcutaneous injection of 100 µg MOG_35–55_ with or without 2 mg GA emulsified in CFA and followed by intraperitoneal injection of B pertussis toxin following emulsion injection and on day 2. On day 20, splenocytes were isolated and stained with anti-CD11b, anti-MHCII and anti-F4/80 antibodies (BD Biosciences) and analyzed by flow cytometry.

### *In vivo* Induction of Type 2 Monocytes

Mice were treated with daily subcutaneous injections of GA (150 µg) for six days. Splenocytes were isolated and CD11c^-^CD11b^+^ cells were purified (>90%) with CD11c and CD11b microbeads (Miltenyi Biotec). Cells were resuspended at 1×10^6^ cells/ml in complete media and treated with 0, 10, or 100 U IFN-γ (Sigma Aldrich) for 48 (TNF-α), 72 (IL-12p40), and 120 (IL-10) hours. Cytokines were analyzed by ELISA (eBioscience).

### *In vitro* Cell-mediated Cytotoxicity Assay

Effector CD8 T cells were derived from *in vitro* stimulated splenocytes and draining lymph nodes of GA-immunized mice as described above. Antigen-loaded ConA blast targets were derived from CFSE-stained overnight cultures of naïve splenocytes incubated with ConA (0.5 µg/mL) in the presence or absence of GA (20 µg/mL). Target cells (T) were plated in a 96-well U-bottom plate at 1000 cells/well in triplicate for each condition. Effectors were added at E:T ratios of 0∶1; 1∶1; 4∶1; 16∶1; 64∶1 in a final volume of 0.2 mL per well. The plates were incubated for 24 hours before fluorescent allophycocyanin beads (APC beads, BD Biosciences) are added as an external number control. The cells were assayed on a BD FACSCalibur flow cytometer. The number of live, CFSE bright cells was quantified and normalized to the number of APC beads in each tube. % Killing was calculated as:
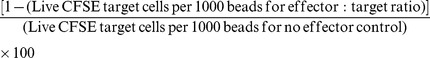



Positive control was redirected lysis of P815 cells. CFSE-stained P815 cells are incubated with anti-CD3 (1 µg/mL) and CD8+ T cells were added at the indicated ratios.

## Supporting Information

Figure S1 **GA-reactive CD8+ T cells express IFN-γ, IL-10, and TNF-α.** Twenty days post-GA/IFA immunization, splenic CD8+ T cells were purified by magnetic bead selection. CD8+ T cells were incubated with irradiated naïve APCs with no antigen, GA or ConA (1 µg/ml). Supernatants were assayed for IFN-γ and IL-10 at 72 hours (A and B). RNA was extracted from *in vitro* activated and purified CD8+ T cells and quantified by real time PCR for IFN-γ, TNF-α, IL-10, IL-6, and TGF-β expression (normalized to actin, C).
(TIF)

Figure S2 **Phagocyte depletion does not affect GA-mediated amelioration of EAE.** (A) C57BL/6 mice were intraperitoneally injected with PBS- or clodronate-loaded liposomes, beginning on day -4 and repeated every 3 days. Disease was induced by subcutaneous injection of emulsion containing MOG_35–55_ (200 µg) and GA (1 mg) or PBS in CFA, followed by intraperitoneal injection of pertussis toxin on day 0 and 2. (B) Splenocytes were isolated from mice in (A) on day 15 and stained with MHCII-Alexa Fluor 700, F4/80-Alexa Fluor 647, and CD11b-PE then analyzed by flow cytometry.
(TIF)

Figure S3 **Splenic monocytes from wild-type and CD8^−/−^ mice acquire a similar degree of anti-inflammatory phenotype after GA treatment** ***in vivo*** **.** Wild-type and CD8^−/−^ mice were subcutaneously injected with GA (150 µg) or PBS daily for 6 days. Splenocytes were isolated and cultured *in vitro* with IFN-γ. Supernatant cytokines were analyzed by ELISA after 48 (TNF-α), 72 (IL-12), and 120 (IL-10) hrs.
(TIF)
