# Supplementary figures and images for: CD8+ T Cells Are Required For Glatiramer Acetate Therapy in Autoimmune Demyelinating Disease
Source: PLoS One. 2013 Jun 21;8(6):e66772. doi: 10.1371/journal.pone.0066772 (PMC3689655; doi:10.1371/journal.pone.0066772)

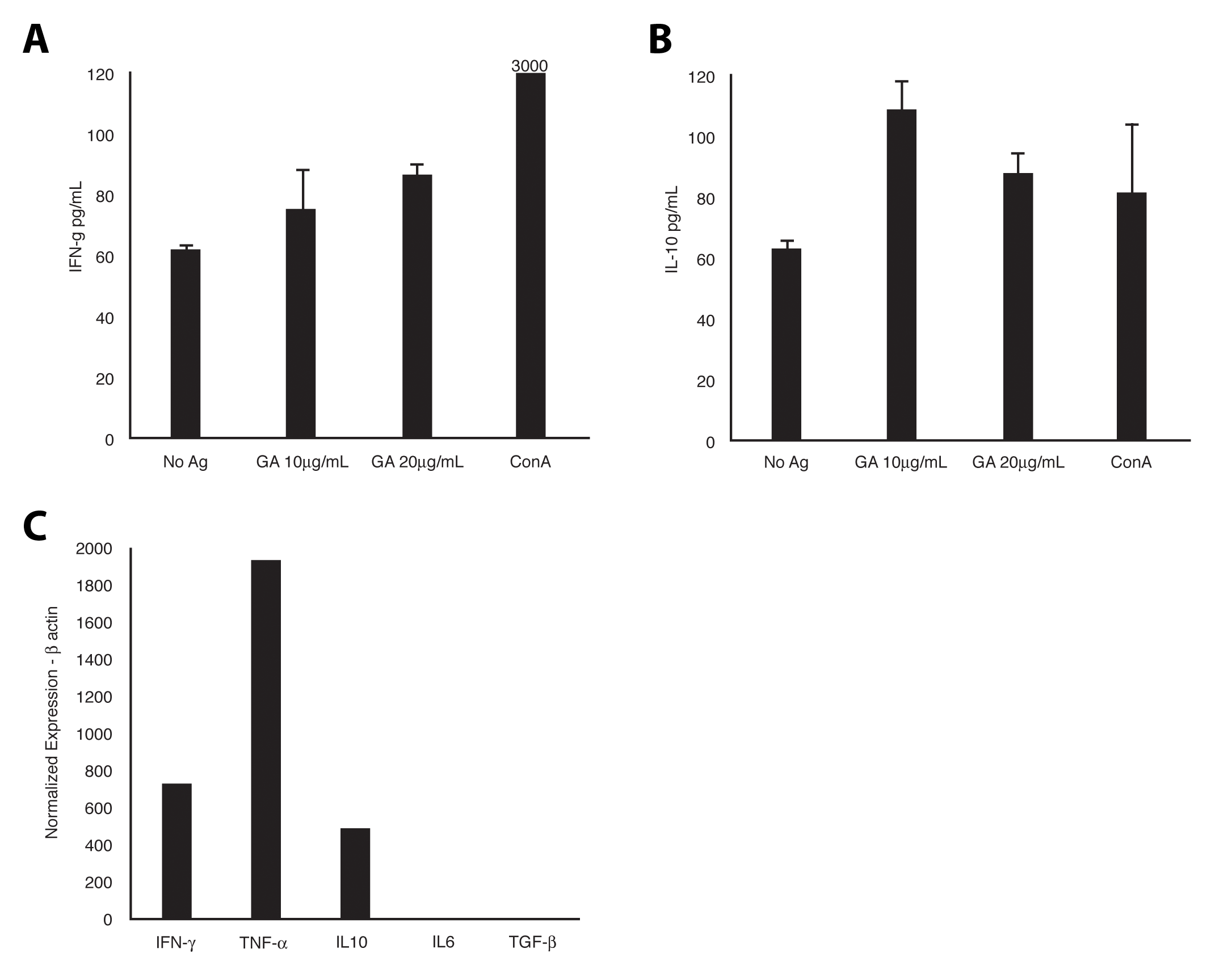

Supplement: Figure S1 — GA-reactive CD8+ T cells express IFN-γ, IL-10, and TNF-α. Twenty days post-GA/IFA immunization, splenic CD8+ T cells were purified by magnetic bead selection. CD8+ T cells were incubated with irradiated naïve APCs with no antigen, GA or ConA (1 µg/ml). Supernatants were assayed for IFN-γ and IL-10 at 72 hours (A and B). RNA was extracted from in vitro activated and purified CD8+ T cells and quantified by real time PCR for IFN-γ, TNF-α, IL-10, IL-6, and TGF-β expression (normalized to actin, C). (TIF) [file pone.0066772.s001.tif]

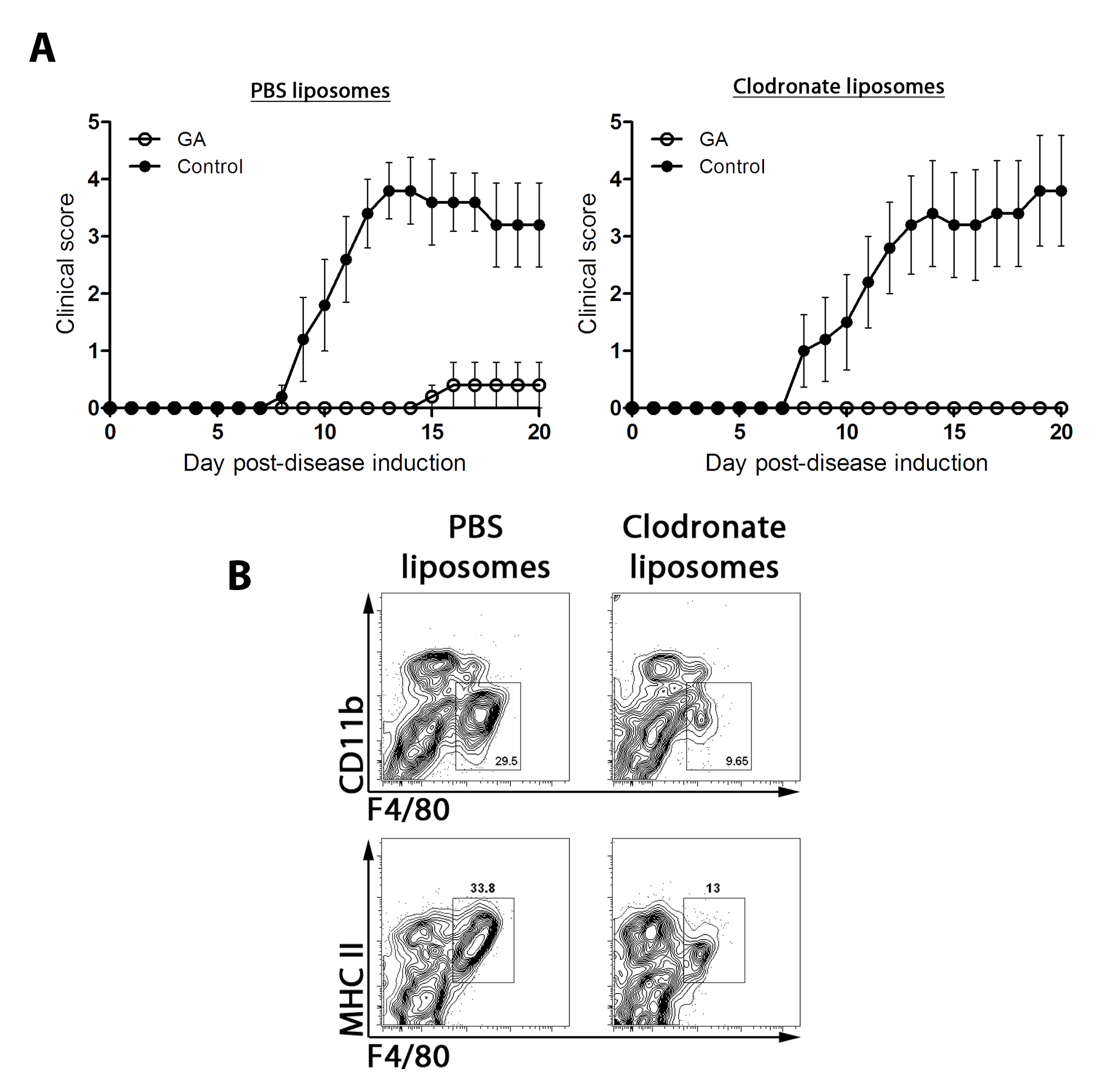

Supplement: Figure S2 — Phagocyte depletion does not affect GA-mediated amelioration of EAE. (A) C57BL/6 mice were intraperitoneally injected with PBS- or clodronate-loaded liposomes, beginning on day -4 and repeated every 3 days. Disease was induced by subcutaneous injection of emulsion containing MOG35–55 (200 µg) and GA (1 mg) or PBS in CFA, followed by intraperitoneal injection of pertussis toxin on day 0 and 2. (B) Splenocytes were isolated from mice in (A) on day 15 and stained with MHCII-Alexa Fluor 700, F4/80-Alexa Fluor 647, and CD11b-PE then analyzed by flow cytometry. (TIF) [file pone.0066772.s002.tif]

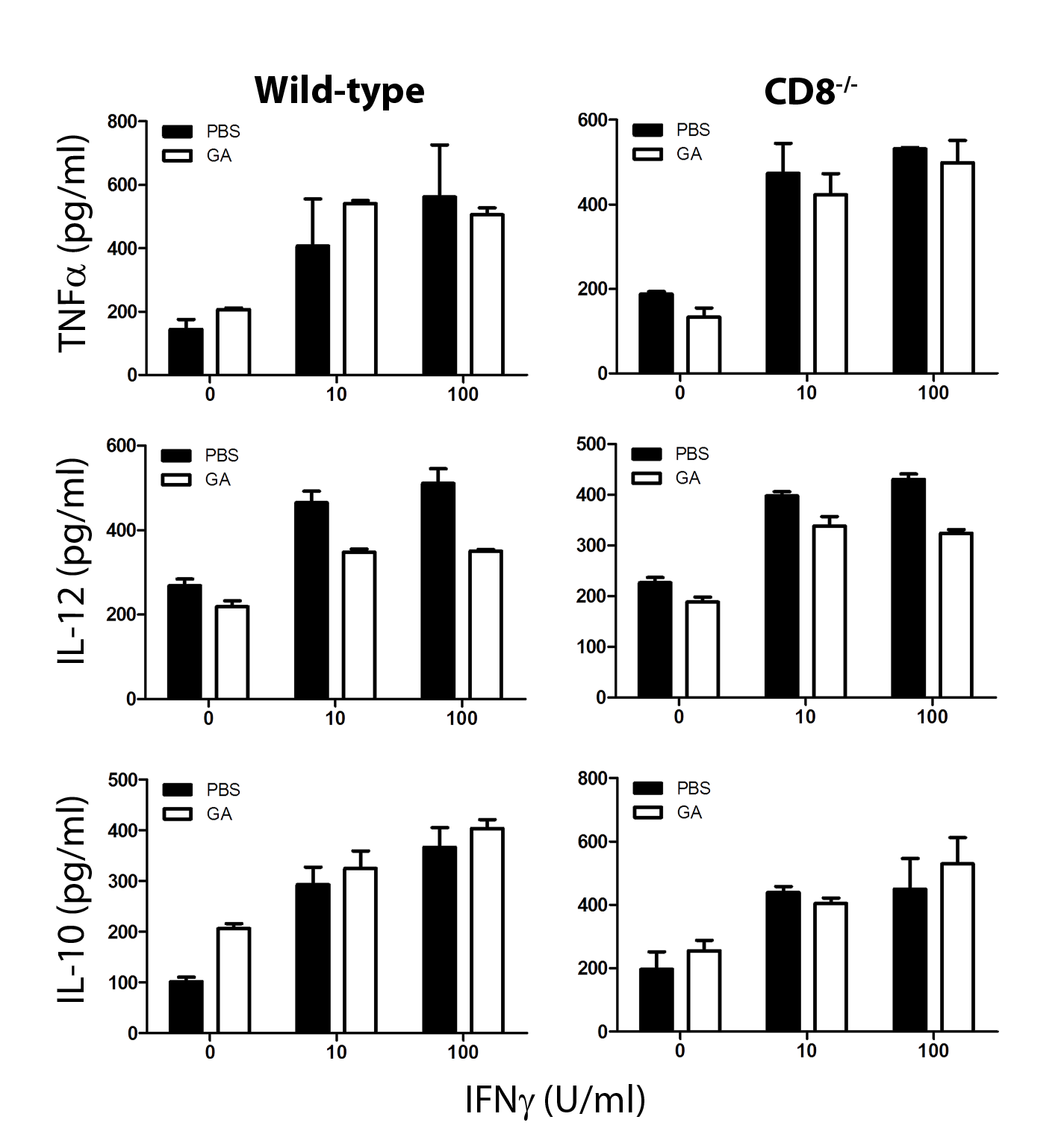

Supplement: Figure S3 — Splenic monocytes from wild-type and CD8−/− mice acquire a similar degree of anti-inflammatory phenotype after GA treatment in vivo . Wild-type and CD8−/− mice were subcutaneously injected with GA (150 µg) or PBS daily for 6 days. Splenocytes were isolated and cultured in vitro with IFN-γ. Supernatant cytokines were analyzed by ELISA after 48 (TNF-α), 72 (IL-12), and 120 (IL-10) hrs. (TIF) [file pone.0066772.s003.tif]
